# RBC balanced immuno-inflammatory signatures identify advanced breast cancer patients on CDK4/6 inhibitors at increased risk of progression and death

**DOI:** 10.1016/j.isci.2025.112620

**Published:** 2025-05-09

**Authors:** Jiayi Ma, Yaohui Wang, Ziping Wu, Liheng Zhou, Yanping Lin, Shuguang Xu, Jie Zhang, Jingsong Lu, Wenjin Yin

**Affiliations:** 1 Department of Breast Surgery, Renji Hospital, School of Medicine, Shanghai Jiao Tong University, Shanghai, China

**Keywords:** Cancer, Metabolomics

## Abstract

The association of immuno-inflammatory parameters, especially RBC balanced signatures, with survival outcomes and adverse events still require investigation for advanced breast cancer (ABC) patients receiving cyclin-dependent kinase 4/6 inhibitor (CDKI). Herein, RBC balanced immuno-inflammatory (RBC-IMM) score was developed and capable of predicting progression-free survival (PFS) events (*p* < 0.001), death (*p* < 0.001) and grade 3/4 leukopenia (*p* = 0.010). RBC-IMM score also predicted PFS more accurately than classical-IMM score (AUC = 0.766 and 0.596 respectively, *p* = 0.005). Besides, clinico+RBC_index exhibited superior performance to clinico_index for 18-month PFS through machine learning (training set: AUC = 0.830 and 0.764 respectively; testing set: AUC = 0.894 and 0.715 respectively). Additionally, liquid chromatography-tandem mass spectrometry identified phosphatidylcholine notably involved in RBC-CDKI interaction, contributing to the construction of clinico+PtdCho_index with better PFS prediction than clinico_index (AUC = 0.854 and 0.733 respectively). These findings indicate that RBC-IMM related parameters have the advantage of identifying benefit and safety in CDKI-treated ABC patients over classical indicators.

## Highlights

• RBC-IMM score is calculated by the count of classical immunocytes balanced by RBC
• RBC-IMM score predicts prognosis and toxicity in ABC patients on CDK4/6 inhibitor
• Phosphatidylcholine may be involved in the interplay between RBC and CDK4/6 inhibitor

## Graphical abstract

**Figure:**
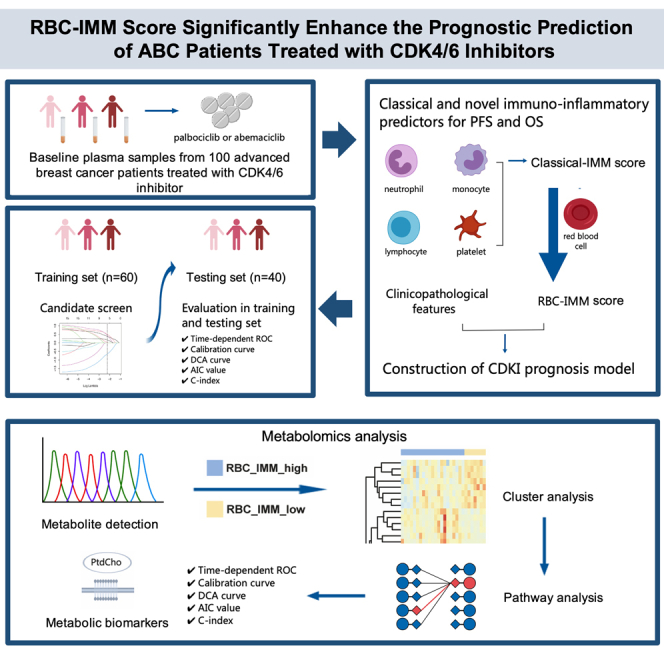

## Introduction

Cyclin-dependent kinases (CDKs) are crucial regulators of cell cycle progression. During the transition from G1 phase to S phase, the inhibition of CDK4/6 impedes the phosphorylation of the Rb protein, preventing the dissociation of transcription factor E2F from Rb protein, thus causing the suppression of genes in the subsequent cascade. Consequently, the cell cycle is arrested at the G1 phase, thereby effectively halting cellular proliferation.^1^^,^^2^^,^^3^ CDK4/6 inhibitor (CDKI) combined with endocrine therapy is recommended as the first line regimen for patients with hormone receptor-positive, human epidermal growth factor receptor 2 (HER2)-negative advanced breast cancer (ABC) according to the current guidelines,^4^^,^^5^ since the series of PALOMA,^6^^,^^7^^,^^8^ MONALEESA,^9^^,^^10^^,^^11^ and MONARCH^12^^,^^13^ clinical trials have demonstrated that the addition of endocrine therapy to CDKI achieved a substantially incremental improvement in progression-free survival (PFS) for these patients. Nevertheless, it is still difficult for physicians to early predict the efficacy of CDKIs.

Diverse opinions currently exist regarding prognosis-related biomarkers. For instance, in PALOMA-3 trial, cyclin E1 expression was found to be associated with resistance to palbociclib when combined with fulvestrant, resulting in reduced antiproliferative effects.^14^ In PALOMA-2 trial, low expression of programmed death ligand 1 (PD-L1) was found to be correlated with the benefit from palbociclib treatment, while tumors with activated growth factor pathways (e.g., FGFR2 and ERBB3) could profit significantly from palbociclib treatment.^15^ By contrast, whole-exome sequencing performed in 59 tumor tissues identified altered activity in genes such as AKT1, RAS, AURKA, CCNE2, ERBB2, and FGFR2 as well as deletion of estrogen receptor, all of which were associated with CDKI resistance.^16^ Notably, these findings have exhibited heterogeneity and can only be delineated in a subset of altered genes. Furthermore, tumor tissue gene sequencing is not so easily accessible that the utilization of liquid biopsies as a source of biomarkers deserve further exploration.

In 1863, the German pathologist Rudolf Virchow proposed the theory that tumor may derive from chronic inflammation. Since then, an increasing number of studies have revealed the interactions between systematic inflammation and tumor.^17^ As important effector cells of intrinsic immunity, neutrophils, lymphocytes, and monocytes play key roles in the systemic and local (tumor microenvironment) immuno-inflammatory status, which have been confirmed to contribute to tumor development as well as the efficacy of antineoplastic therapy.^18^ Previous studies have reported that the absolute peripheral blood lymphocyte (ALC), neutrophil to lymphocyte ratio (NLR), lymphocyte to monocyte ratio (LMR), and platelet to lymphocyte ratio (PLR) can serve as prognostic predictors for solid tumors.^19^^,^^20^^,^^21^ For patients with HER2-negative ABC treated with paclitaxel plus bevacizumab, high ALC, low NLR, high LMR, and low PLR could predict longer overall survival (OS).^22^ Additionally, red blood cells (RBCs) have also been found involved in organismal immunity.^23^^,^^24^ Our previous study first used RBC as a calibration modality to calculate the lymphocyte to RBC ratio (LRR), neutrophil to RBC ratio (NRR), and monocyte to RBC ratio (MRR) in locally advanced breast cancer treated with neoadjuvant therapy, and revealed that LRR-NRR-MRR could be an independent predictor of disease-free survival (DFS) and OS for these patients.^25^ Subsequent study by Grupinska et al. further confirmed these findings in early breast cancer.^26^ However, no study has reported the performance of RBC balanced immuno-inflammatory biomarkers in predicting the efficacy of CDKIs or in other subtypes of ABC.

Tumors are characterized by metabolic reprogramming, where tumor cells adapt to extrinsic and intrinsic stress conditions such as hypoxia, acidity, and nutrient deficiency by enhancing glycolysis, tricarboxylic acid cycle activity, glutaminolysis, and fatty acid biosynthesis. This transformation of energy metabolism pathways allows for rapid adaptation to meet the needs of cell proliferation.^27^^,^^28^^,^^29^^,^^30^ Liquid chromatography-tandem mass spectrometry (LC-MS/MS) enables fingerprint analysis of metabolites. By comparing the mass peaks of metabolic products to study their characteristic patterns, differentially expressed substances can be discovered, which can aid in the search of predictive biomarkers for diseases^31^^,^^32^ and predictive substances for drug response.^33^^,^^34^ As to breast cancer, Wei et al. performed metabolomics study in 28 patients treated with neoadjuvant chemotherapy and elucidated that patients with high levels of isoleucine and low levels of threonine and glutamine before chemotherapy were more likely to achieve pathological complete response.^35^ However, little is known about the value of metabolomics in predicting CDKI efficacy. Wang et al. found in cellular experiments that fructose-2,6-bisphosphatase 4 promotes glycolysis, enhances cellular stemness, and triggers metabolic reprogramming, making hormone receptor-positive breast cancer cells resistant to palbociclib.^36^ Marangoni et al. discovered through patient-derived xenograft mouse models that elevated levels of oxidative phosphorylation are associated with resistance to palbociclib in combination with endocrine therapy.^37^ Therefore, clinical studies are warranted for further exploration and validation.

Accordingly, we aimed to explore the role of baseline LRR, NRR, MRR, and PRR, as well as baseline NLR, LMR, and PLR, in assessing the prognosis of ABC patients treated with CDKIs. We also constructed an RBC balanced immuno-inflammatory (RBC-IMM) score and created a comprehensive index, integrating clinicopathological parameters and RBC-IMM score, to predict the survival outcomes and adverse events (AEs) of CDKI. Furthermore, we carried out metabolomics to discover the potential mechanisms whereby RBC-related immuno-inflammatory parameters may predict the efficacy and toxicity of CDKI.

## Results

### Patient characteristics

A total of 100 Chinese female patients were included in this study, of which 50% received palbociclib and the rest were treated with abemaciclib. Pre-menopausal patients accounted for 28%, and 30% were >65 years old. At baseline, 52% of all patients had at least two sites of metastasis. Liver involvement was documented in 39% of patients. Besides, 42% received CDKI as first-line therapy for their advanced diseases and 31% were administered second-line CDKI. Before the application of CDKI, 45% of the enrolled patients received chemotherapy in the advanced setting. No statistical differences were observed between palbociclib group and abemaciclib group in terms of baseline characteristics (Table 1).

**Table 1:** Clinicopathological characteristics in all patients and patients receiving different CDKIs

Characteristics	Total *N* = 100 (%)	Palbociclib *N* = 50 (%)	Abemaciclib *N* = 50 (%)	*p* value
Age of starting CDKI				
<65	70 (70)	38 (76)	32 (64)	0.19
≥65	30 (30)	12 (24)	18 (36)	
Menopausal status				
Pre-menopausal	28 (28)	12 (24)	16 (32)	0.37
Post-menopausal	72 (72)	38 (76)	34 (68)	
Visceral involvement				
Yes	69 (69)	35 (70)	34 (68)	0.83
No	31 (31)	15 (30)	16 (32)	
Liver involvement				
Yes	39 (39)	22 (44)	17 (34)	0.31
No	61 (61)	28 (56)	33 (56)	
Number of metastatic sites				
≤1	48 (48)	19 (42)	18 (47)	0.64
>1	52 (52)	26 (58)	20 (53)	
Line of CDKI for advanced disease				
1^st^	42 (42)	23 (46)	19 (38)	0.51
2^nd^	31 (31)	16 (32)	15 (30)	
≥3^rd^	27 (27)	11 (22)	16 (32)	
Previous chemotherapy for advanced disease				
Yes	45 (45)	20 (40)	25 (50)	0.31
No	55 (55)	30 (60)	25 (50)	

Abbreviations: CDKI, cyclin-dependent kinases 4/6 inhibitor.

### Univariate and multivariate analysis of PFS and OS

The median follow-up interval was 19.60 months (95% confidence intervals [CI], 16.93–22.37 months) for the whole cohort, 25.63 months (95% CI 23.33–31.40 months) for palbociclib group, and 14.50 months (95% CI 11.80–16.93 months) for abemaciclib group. A total of 73 patients (73%) progressed or died during CDKI treatment with the median PFS (mPFS) of 11.37 months (95% CI 8.90–14.07 months) for all the enrolled patients, 9.20 months (95% CI 5.67–13.53 months) for palbociclib group and 11.50 months (95% CI 9.90–15.30 months) for abemaciclib group.

**Figure 1:**
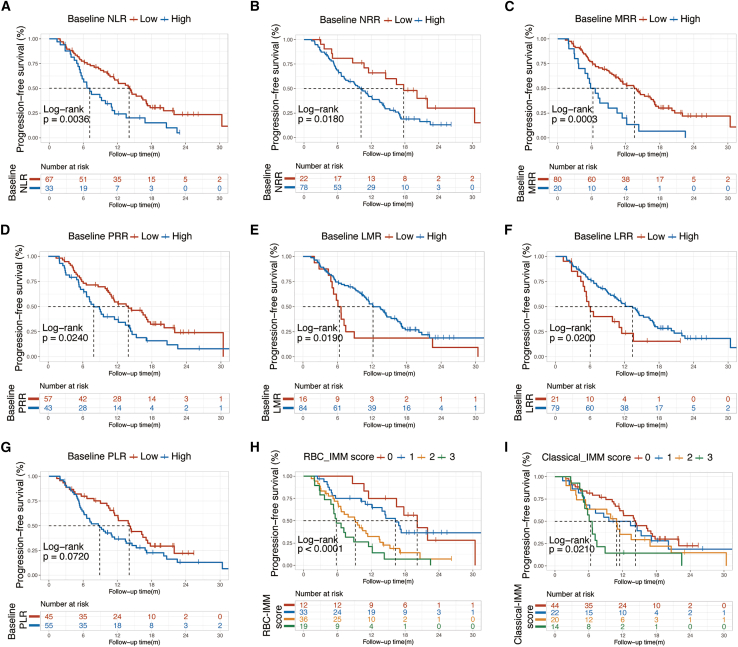
Progression-free survival by different immuno-inflammatory parameters Kaplan–Maier analysis of progression-free survival in all patients treated with CDKI by baseline (A) NLR (high vs. low), (B) NRR (high vs. low), (C) MRR (high vs. low), (D) PRR (high vs. low), (E) LMR (high vs. low), (F) LRR (high vs. low), (G) PLR (high vs. low), (H) RBC-IMM score (3 vs. 2 vs. 1 vs. 0), and (I) classical-IMM score (3 vs. 2 vs. 1 vs. 0). Abbreviations: NLR, neutrophil to lymphocyte ratio; LMR, lymphocyte to monocyte ratio; PLR, platelet to lymphocyte ratio; LRR, lymphocyte to red blood cell ratio; NRR, neutrophil to red blood cell ratio; MRR, monocyte to red blood cell ratio; PRR, platelet red blood cell ratio; RBC, red blood cell; IMM, immuno-inflammatory; CDKI, cyclin-dependent kinases 4/6 inhibitor.

The univariate analysis of PFS in all patients (Table 2) showed that mPFS was shorter in the high level group than that in the low level group for NLR (Figure 1A), NRR (Figure 1B), MRR (Figure 1C) and PRR (Figure 1D), while the opposite trend was observed in LMR (Figure 1E) and LRR (Figure 1F). The high PLR group tended to have inferior mPFS to the low group (Figure 1G). Moreover, patients with higher RBC-IMM score were more likely to experience disease progression or death (Figure 1H) and classical-IMM score displayed the same trend (Figure 1I).

**Table 2:** Univariate Cox analysis of PFS in all patients

Characteristics	Comparison	HR (95% CI)	*p* value (FDR)
Age	≥65 vs. <65	0.50 (0.29–0.85)	**0.010 (0.023)**
Menopausal status	Post- vs. pre-	0.58 (0.35–0.95)	**0.032 (0.042)**
Visceral involvement	Yes vs. No	1.69 (1.00–2.86)	**0.049 (0.052)**
Liver involvement	Yes vs. No	1.97 (1.24–3.15)	**0.004 (0.011)**
Number of metastatic sites	>1 vs. 1	1.66 (1.04–2.64)	**0.034 (0.042)**
Line of CDKI treatment	>1^st^ vs. 1^st^	2.23 (1.34–3.71)	**0.002 (0.011)**
Previous chemotherapy for advanced disease	Yes vs. No	1.60 (1.00–2.55)	**0.049 (0.052)**
Baseline NLR	High vs. Low	2.01 (1.24–3.25)	**0.004 (0.011)**
Baseline LMR	High vs. Low	0.50 (0.28–0.90)	**0.021 (0.033)**
Baseline PLR	High vs. Low	1.54 (0.96–2.47)	0.074 (0.074)
Baseline LRR	High vs. Low	0.52 (0.29–0.91)	**0.023 (0.033)**
Baseline NRR	High vs. Low	1.98 (1.10–3.56)	**0.023 (0.033)**
Baseline MRR	High vs. Low	2.60 (1.52–4.45)	**<0.001 (<0.001)**
Baseline PRR	High vs. Low	1.73 (1.09–2.75)	**0.020 (0.033)**
Baseline classical-IMM score	3 vs. 2 vs. 1 vs. 0	1.36 (1.10–1.68)	**0.004 (0.011)**
Baseline RBC-IMM score	3 vs. 2 vs. 1 vs. 0	1.80 (1.39–2.33)	**<0.001 (<0.001)**

Abbreviations: PFS, progression-free survival; CDKI, cyclin-dependent kinases 4/6 inhibitor; NLR, neutrophil to lymphocyte ratio; LMR, lymphocyte to monocyte ratio; PLR, platelet to lymphocyte ratio; LRR, lymphocyte to red blood cell ratio; NRR, neutrophil to red blood cell ratio; MRR, monocyte to red blood cell ratio; PRR, platelet red blood cell ratio; RBC, red blood cell; IMM, immuno-inflammatory; HR, hazard ratio; CI, confidence interval; FDR, false discovery rate.

Univariate analyses of PFS for palbociclib and abemaciclib groups (Table S1) revealed the same tendency to the whole cohort. Higher RBC-IMM score also predicted poorer PFS in the two groups (unadjusted *p* = 0.003 and *p* = 0.003), while high classical-IMM score predicted poorer PFS in abemaciclib (unadjusted *p* = 0.019) group rather than in palbociclib group (unadjusted *p* = 0.136). Similar effects of RBC-IMM and classical-IMM scores on PFS were observed in different lines of CDKI for advanced disease (Table S2).

The multivariate analysis of PFS for the whole patients (Table 3) showed that patients aged ≥65 years (*p* = 0.001) and those receiving CDKI as first line therapy for advanced disease (*p* = 0.013) were less likely to experience PFS event. RBC-IMM score (*p* = 0.003), rather than classical-IMM score (*p* = 0.337), was an independent detrimental factor for disease progression or death during CDKI therapy.

**Table 3:** Multivariate analysis of PFS in all patients

Characteristics	HR (95% CI)	*p* value (FDR)
Age		
≥65 vs. <65	0.40 (0.23–0.70)	**0.001 (0.005)**
Visceral involvement		
Yes vs. No	1.56 (0.91–2.69)	0.107 (0.107)
Line of CDKI treatment		
1^st^ vs. >1^st^	0.52 (0.31–0.87)	**0.013 (0.021)**
Baseline classical-IMM score		
3 vs. 2 vs. 1 vs. 0	1.13 (0.88–1.47)	0.337 (0.337)
Baseline RBC-IMM score		
3 vs. 2 vs. 1 vs. 0	1.63 (1.19–2.24)	**0.003 (0.008)**

Abbreviations: PFS, progression-free survival; CDKI, cyclin-dependent kinases 4/6 inhibitor; RBC, red blood cell; IMM, immuno-inflammatory; FDR, false discovery rate.

**Figure 2:**
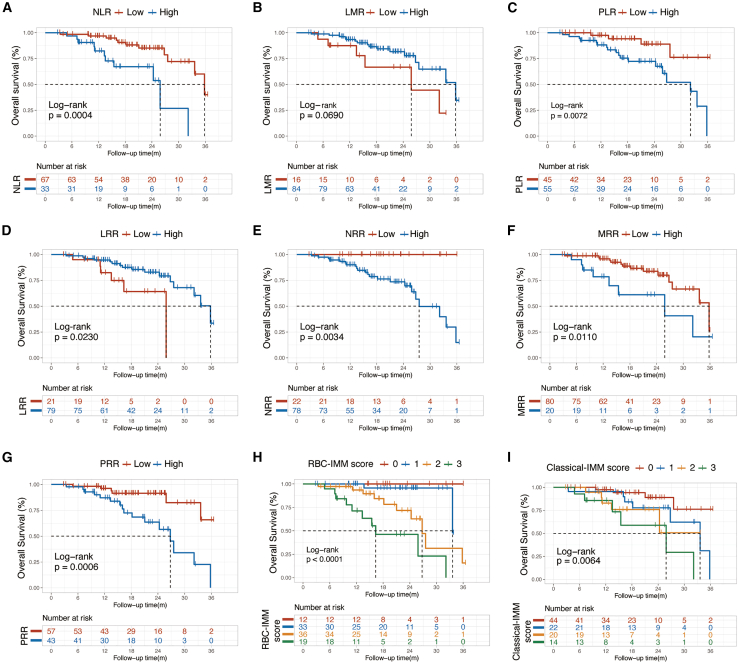
Overall survival by different immuno-inflammatory parameters Kaplan–Maier analysis of overall survival in all patients treated with CDKI by baseline (A) NLR (high vs. low), (B) LMR (high vs. low), (C) PLR (high vs. low), (D) LRR (high vs. low), (E) NRR (high vs. low), (F) MRR (high vs. low), (G) PRR (high vs. low), (H) RBC-IMM score (3 vs. 2 vs. 1 vs. 0) and (I) classical-IMM score (3 vs. 2 vs. 1 vs. 0). Abbreviations: NLR, neutrophil to lymphocyte ratio; LMR, lymphocyte to monocyte ratio; PLR, platelet to lymphocyte ratio; LRR, lymphocyte to red blood cell ratio; NRR, neutrophil to red blood cell ratio; MRR, monocyte to red blood cell ratio; PRR, platelet red blood cell ratio; RBC, red blood cell; IMM, immuno-inflammatory; CDKI, cyclin-dependent kinases 4/6 inhibitor.

A total of 22 patients died (22%). The median OS (mOS) was 33.60 months (95% CI 26.77-not estimable) for all patients and 32.13 months for the palbociclib group (95% CI 25.83-not estimable). OS in abemaciclib group was immature. The univariate analysis of OS in all patients (Table S3) showed the same trend as PFS for NLR (Figure 2A), LMR (Figure 2B), PLR (Figure 2C), LRR (Figure 2D), NRR (Figure 2E), MRR (Figure 2F), and PRR (Figure 2G). Patients with high RBC-IMM score (Figure 2H) or those with high classical-IMM score (Figure 2I) were more likely to face an incremental risk of death during CDKI treatment. The multivariate analysis revealed that RBC-IMM score, instead of classical-IMM score, was an independent prognosticator for OS (*p* = 0.001; Table S4).

### Establishment of predictive model for PFS

**Figure 3:**
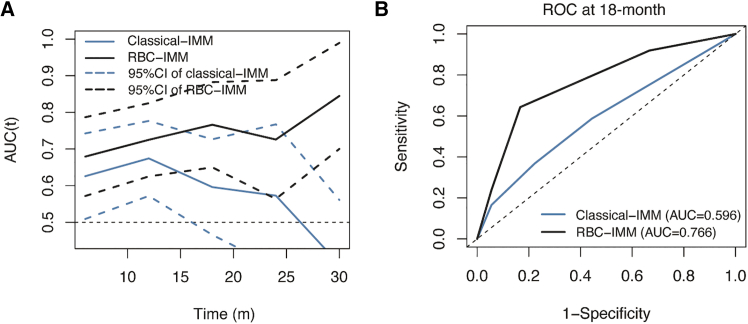
Time-dependent ROC curve analysis to predict the accuracy of different scores for PFS after initiating CDKIs (A) Time-dependent AUC and 95%CI of the classical-IMM and RBC-IMM scores. (B) ROC curves at 18-month of the classical-IMM and RBC-IMM scores. Abbreviations: ROC, receiver operating characteristic; PFS, progression-free survival; AUC, area under the curve; CI, confidence interval; RBC, red blood cell; IMM, immuno-inflammatory; CDKI, cyclin-dependent kinases 4/6 inhibitor.

We applied a time-dependent receiver operating characteristic (ROC) analysis to compare the performance of RBC-IMM and classical-IMM scores (Figure 3A). As a result, RBC-IMM score predicted the 18-month PFS more accurately than classical-IMM score, with the area under the curve (AUC) value of 0.766 and 0.596, respectively (*p* = 0.005; Figure 3B). Akaike information criterion (AIC) values and Harrell’s concordance index (C-index) showed the similar tendency, with AIC values of 549.76 and 562.38, and C-index of 0.66 and 0.60, for RBC-IMM and classical-IMM scores respectively.

**Figure 4:**
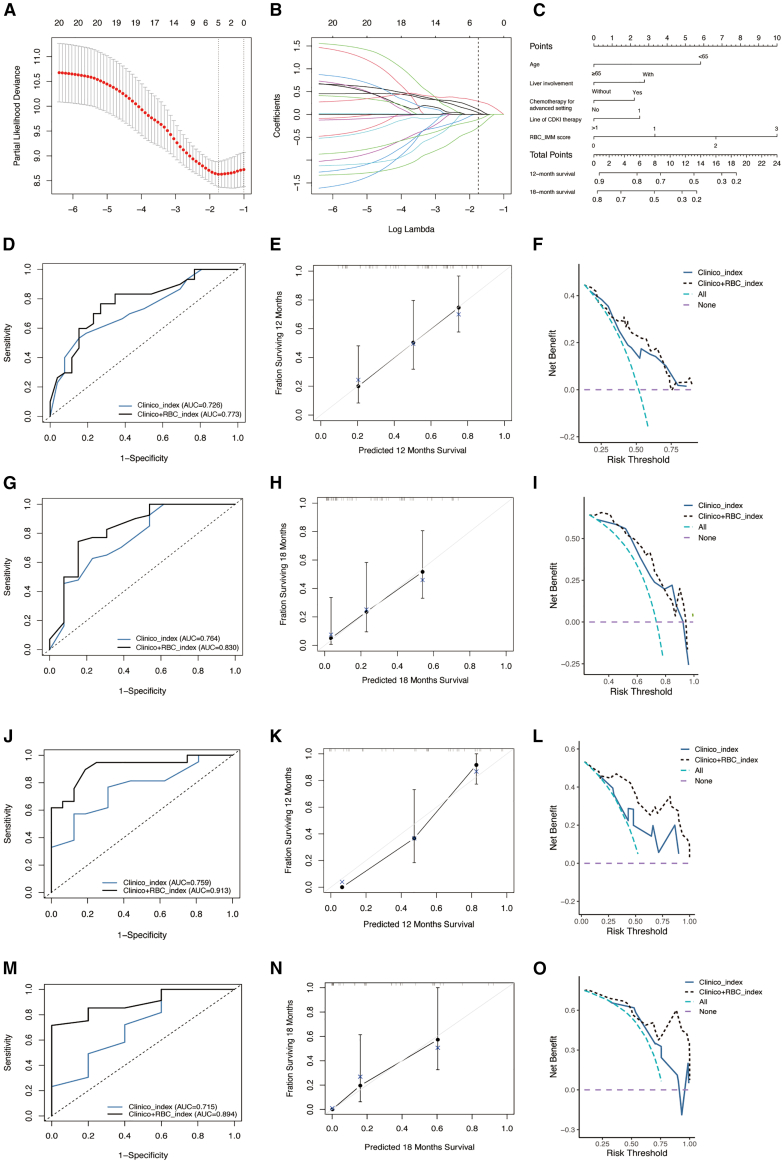
Model building and assessment for progression-free survival (A) LASSO algorithm and 10-fold cross validation for feature selection. (B) The top five variables, including RBC-IMM score, age, liver involvement, chemotherapy for advanced disease and line of CDKI treatment, were extracted with λ = 0.174 [log(λ) = −0.760]. LASSO coefficient profiles of candidate features were successively selected into the predictive model. (C) Nomogram based on clinico + RBC_index in training set. (D and G) ROC curves of clinico_index model (blue) and clinico + RBC_index model (black) at 12-month (D) and 18-month (G) for training set. (E and H) Calibration curves of clinico + RBC_index at 12-month (E) and 18-month (H) in training set. (F and I) Decision curve analysis for net clinical benefit of clinico_index model (blue line) and clinico + RBC_index (black line) at 12-month (F) and 18-month (I) in training set. (J and M) ROC curves of clinico_index model (blue) and clinico + RBC_index model (black) at 12-month (J) and 18-month (M) for testing set. (K and N) Calibration curves of clinico + RBC-IMM_index at 12-month (K) and 18-month (N) in testing set. (L and O) Decision curve analysis for net clinical benefit of clinico_index model (blue line) and clinico + RBC_index model (black line) at 12-month (L) and 18-month (O) in testing set. Abbreviations: CDKI, cyclin-dependent kinases 4/6 inhibitor; RBC, red blood cell; IMM, immuno-inflammatory; ROC, receiver operating characteristic curves; AUC, area under the curve; LASSO, least absolute shrinkage and selection operator.

To optimize the predictive performance for PFS, the enrolled patients were randomized into a training set and a testing set at a 6:4 ratio. Twenty candidate variables were screened through least absolute shrinkage and selection operator (LASSO) regression algorithm and cross validation (Figure 4A). Then the top five variables were selected, including age, liver involvement, RBC-IMM score, chemotherapy for advanced disease, and line of CDKI (Figure 4B). The clinico+RBC_index was composed of all these five variables, while the clinico_index incorporated age, liver involvement, chemotherapy for advanced disease, and line of CDKI. A nomogram was created for the clinico+RBC_index (Figure 4C). The ROC curve confirmed the superior accuracy of the clinico+RBC_index over clinico_index. As a result, the AUC value in the training set was 0.773 for clinico+RBC_index and 0.726 for clinico_index at 12 months after initiating CDKI (Figure 4D), along with 0.830 and 0.764 respectively at 18-month (Figure 4G). The corresponding calibration curves indicated that the predicted probabilities of PFS were consistent with the observed outcomes for both 12-month and 18-month PFS (Figures 4E and 4H). The decision curve analysis (DCA) curves also depicted the net benefit of the clinico+RBC_index when compared with the clinico_index (Figures 4F and 4I). For the testing set, both the ROC (AUC = 0.913 and 0.759 at 12-month, Figure 4J; AUC = 0.894 and 0.715 at 18-month; Figure 4M) curves and the calibration curve validated the better performance of clinico+RBC_index than clinico_index (Figures 4K and 4N). DCA analysis also verified the improvement of adding RBC-IMM score to clinicopathological features (Figures 4L and 4O). Besides, the AIC values were 100.48 and 113.74, and the C-indexes were 0.80 and 0.64 for the index with and without RBC-IMM score, respectively.

### Performance of RBC-IMM and classical-IMM scores in predicting AEs

Safety was assessed in evaluable patients (Table 4). Overall, hematological and biochemical AEs were obtained in 69 (69%) and 68 (68%) patients respectively (Table S5). Higher RBC-IMM score (odds ratio [OR] = 2.50, 95% CI 1.25–5.01, *p* = 0.010) or classical-IMM score (OR = 1.91, 95% CI 1.12–3.27, *p* = 0.018) was associated with grade 3–4 leukopenia. Additionally, increased aspartate aminotransferase (AST) during CDKI application was likely associated with higher RBC-IMM score (OR = 1.94, 95% CI 1.00–3.74, *p* = 0.049). No differences were detected for the other hematological and biochemical AEs.

**Table 4:** Predictive performance of RBC-IMM score and classical-IMM score for adverse events

Characteristics	Comparison for OR	RBC-IMM score (high vs. low)			Classical-IMM score (high vs. low)		
		Univariate OR	95% CI	*p* value	Univariate OR	95% CI	*p* value
Leukopenia	0-2 vs. 3-4	2.50	1.25–5.01	**0.010**	1.91	1.12–3.27	**0.018**
Neutropenia	0-2 vs. 3-4	1.50	0.82–2.75	0.191	1.63	0.98–2.72	0.062
Thrombocytopenia	0 vs. 1-4	0.96	0.55–1.67	0.882	0.64	0.37–1.10	0.105
Anemia	0 vs. 1-4	0.80	0.42–1.51	0.487	0.66	0.35–1.23	0.189
AST increased	0 vs. 1-4	1.94	1.00–3.74	**0.049**	1.32	0.79–2.21	0.287
ALT increased	0 vs. 1-4	1.34	0.63–2.84	0.449	1.00	0.53–1.90	1.000
Blood creatinine level increased	0 vs. 1-4	1.05	0.57–1.93	0.876	1.15	0.69–1.93	0.592

Abbreviations: RBC, red blood cell; IMM, immuno-inflammatory; AST, aspartate aminotransferase; ALT, alanine aminotransferase; OR, odds ratio; CI, confidence interval.

### Correlations of circulating immune cells with immune-inflammatory parameters

To evaluate the systematic impact of the immuno-inflammatory parameters, we investigated their interactions with circulating immunocyte subsets in patients with available data (Figure S1A). The results revealed that LMR was positively correlated with the expression of CD3^+^CD4^+^ T cells (Figure S1B) and negatively correlated with the expression of CD56^+^CD16^+^ natural killer (NK) cells (Figure S1C). Besides, PLR (Figure S1D) and PRR (Figure S1E) were positively correlated with the level of CD56^+^CD16^+^ NK cells, and PRR was positively correlated with the absolute value of NK cells (Figure S1F). Interestingly, RBC-IMM score was positively correlated with CD56^+^CD16^+^ NK cells (Figure S1G). However, no significant correlation was observed between classical-IMM score and immunocyte subsets.

### Metabolomics and KEGG analysis

**Figure 5:**
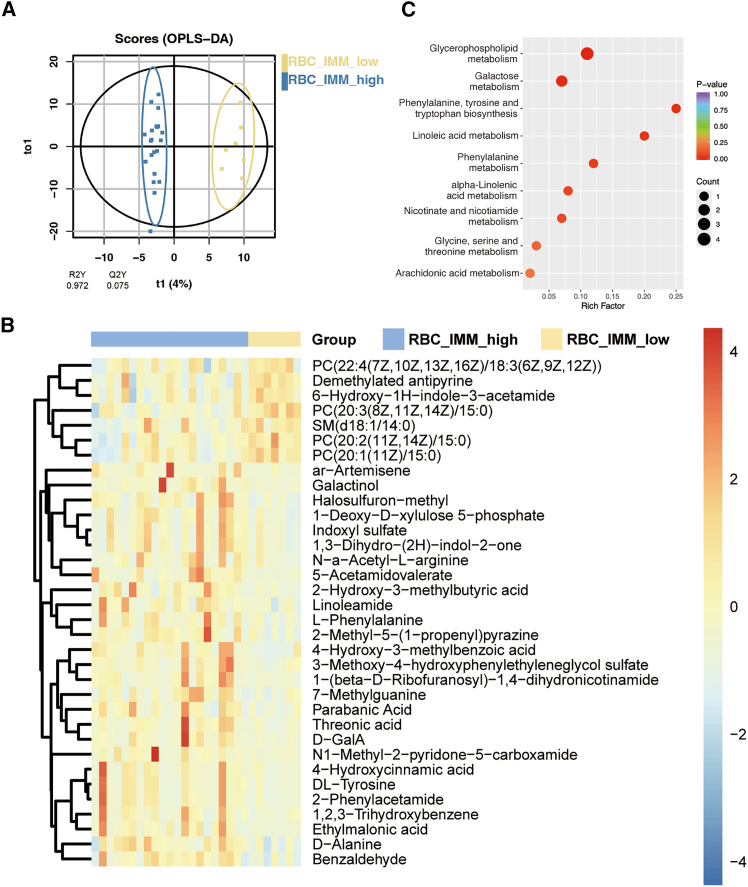
Metabolomics of 28 samples (A) OPLS-DA score plot based on RBC-IMM low-risk (yellow) and high-risk (blue) group. (B) Differentially expressed metabolites clustering heatmap according to RBC-IMM score (high-risk vs. low-risk). Each column represents a sample and each row represents a metabolite. The color indicates the relative expression of metabolites. There is a tree of metabolites clustering on the left with the names of metabolites on the right. (C) Metabolic pathway analysis according to the KEGG based on differentially expressed metabolites. Abbreviations: OPLS-DA, orthogonal projection to latent structures discriminant analysis; RBC, red blood cell; IMM, immuno-inflammatory; KEGG, Kyoto Encyclopedia of Genes and Genomes.

Metabolomics testing was conducted using the serum of 31 patients before CDKI application, among which RBC-IMM score were available in 28 patients. The 28 patients were divided into two groups according to RBC-IMM score, of which 21 were in the high-risk group (score 1–3). Orthogonal projections to latent structures-discriminate analysis (OPLS-DA) analysis of the detected metabolites was performed (Figure 5A), which yielded 34 differential metabolites (Figure 5B; Table S6), including phosphatidylcholine, D-galactose, and L-phenylalanine. We found that these differential metabolites affected all the metabolite categories, mainly distributed in lipids and lipid-like molecules, organoheterocyclic compounds, and organic acids and derivatives according to human metabolome database (HMDB) (Figure S2) and illustrated part of them in the overview of metabolism network using iPath 3 (Figure S3). Network plot displayed their interaction (Figure S4). Through Kyoto Encyclopedia of Genes and Genomes (KEGG) enrichment, we found that metabolites associated with the different levels of RBC-IMM score were most enriched in the glycerophospholipid metabolism pathway (Figure 5C). In this pathway, different structure of phosphatidylcholines all exhibited low expression in RBC-IMM high-risk group (Figure S5).

For the classical-IMM score, we also conducted OPLS-DA score plot (Figure S6A) and differential metabolite analysis (Figure S6B; Table S7). KEGG analysis revealed that the differential metabolites were mainly related to glycerophospholipid metabolism, linoleic acid metabolism, and taurine and hypotaurine metabolism (Figure S6C). Network analysis revealed that the relationship between taurine and creatine was essential to classical-IMM risk grouping (Figure S6D).

### Prognostic performance of phosphatidylcholine related metabolites

**Figure 6:**
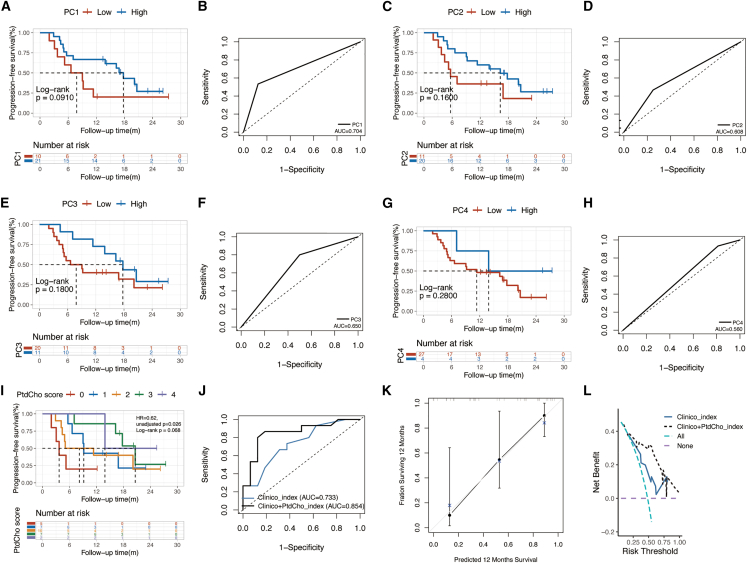
Prognostic performance of each PtdCho and PtdCho score in the metabolic cohort Kaplan–Maier analysis (A, C, E, and G) and ROC curve (B, D, F, and H) at 12-month in terms of progression-free survival for metabolomics cohort by baseline (A and B) PC1 (high vs. low), (C and D) PC2 (high vs. low), (E and F) PC3 (high vs. low) and (G and H) PC4 (high vs. low). (I) Kaplan–Maier analysis of progression-free survival by PtdCho score (4 vs. 3 vs. 2 vs. 1 vs. 0). (J) ROC curves of clinico_index (blue line) and clinico + PtdCho index (black line). (K) Calibration curves of clinico + PtdCho_index at 12-month. (L) Decision curve analysis for net clinical benefit of clinico_index (blue line) and clinico + PtdCho index (black line) at 12-month. Abbreviations: ROC, receiver operating characteristic curves; AUC, area under the curve; PC1, PC(22:4(7Z,10Z,13Z,16Z)/18:3(6Z,9Z,12Z)); PC2, PC(20:3(8Z,11Z,14Z)/15:0); PC3, PC(20:2(11Z,14Z)/15:0); PC4, PC(20:1(11Z)/15:0); PtdCho, phosphatidylcholine.

To translate the metabolomics discoveries into clinical practice, we evaluated the prognostic value of each phosphatidylcholine differentially expressed between the RBC-IMM high- and low-risk group in the metabolic cohort through both Kaplan–Maier analysis and ROC curve at 12 months in terms of PFS for the metabolomics cohort (Figures 6A–6H). Combining the four phosphatidylcholines, we developed a phosphatidylcholine-related integrated indicator (PtdCho score). Univariate analysis showed that higher baseline PtdCho score predicted a lower risk of progression or death after initiating CDKIs (hazard ratio [HR] = 0.62, 95% CI 0.41–0.94, unadjusted *p* = 0.026, Figure 6I). The AUC value of the clinico+PtdCho_index (PtdCho score combined with age, liver involvement, chemotherapy for advanced disease and line of CDKI) was 0.854, while the AUC value of the clinico_index was 0.733 (*p* = 0.083, Figure 6J). The corresponding calibration curves indicated that the predicted probabilities were consistent with the observed outcomes for the 12-month PFS (Figure 6K). The DCA curves displayed the net benefit of the clinico + PtdCho_index compared with the clinico_index (Figure 6L). The C-index was 0.66 and 0.58 for the combined model and the clinicopathological model, and the AIC value was 117.47 and 122.44, respectively. The exploratory analysis indicated that both PC(22:4(7Z,10Z,13Z,16Z)/18:3(6Z,9Z,12Z)) level and PtdCho score were positively related with CD3^+^CD4+T cells (Figure S7A, *p* = 0.032 and *p* = 0.020), but negatively associated with CD3^+^CD8^+^T cells (Figure S7B, *p* = 0.020 and 0.048), while no statistical difference was observed in CD56^+^CD16^+^ NK cells and absolute value of NK cells (Figures S7C and S7D).

## Discussion

In our study, we applied baseline RBC balanced immuno-inflammatory parameters, along with the well-studied markers such as NLR, LMR, and PLR, for the prediction of PFS and OS in hormone receptor-positive, HER2-negative ABC patients treated with CDKI. We also constructed RBC-IMM score, as a novel biomarker reflecting immuno-inflammatory condition of patients, which may outperform the classical-IMM score in predicting both PFS and AEs. Furthermore, our metabolomics analysis demonstrated the positive correlation of immuno-inflammatory metabolites, exemplified by phosphatidylcholines, with RBC-IMM score as well as their prognostic value in the patients treated with CDKI. These remarkable findings may pave the way for a deeper understanding of the complex interplay between immuno-inflammatory factors, especially RBC balanced features or phosphatidylcholine related metabolites, and the efficacy of CDKI therapy in ABC patients.

Previous studies have demonstrated the prognostic significance of NLR, LMR, and PLR as systematic classical immuno-inflammatory factors in liquid biopsy for solid tumors.^19^^,^^20^^,^^21^ In terms of CDKI, a retrospective analysis of PALOMA-2/3 identified that the lower level of NLR and absolute neutrophil/absolute leucocyte-neutrophil values in palbociclib treatment predicted longer PFS,^38^ which was also confirmed in a real-world retrospective study.^39^ However, the relationship of CDKI efficacy with LMR and PLR has not been reported yet in CDKI-treated ABC patients. Our study revealed unfavorable impact of both NLR and PLR on the prognosis, while high level of LMR indicated a longer PFS as well as OS for patients treated with CDKI, particularly abemaciclib. According to previous publications, CDKI can exercise anti-tumor effect by stimulating innate immunity, reducing the proliferation of regulatory T cells,^40^ driving the phenotypic and functional acquisition of immunologic T cell memory and enhancing T cell activation so as to promote the activity of cytotoxic T cell and inflammatory response.^41^^,^^42^ Preclinical studies also found that CDKI combined with immune checkpoint inhibitors could further downregulate cell cycle genes and increase immune memory of tumor cells through Rb protein,^42^ exhibiting synergistic anti-tumor effect.^43^^,^^44^ These lines of evidence indicated that immune cells such as lymphocytes, neutrophils and NK cells are involved in the action of CDKI. Therefore, we developed the immune-related prognostic factors for ABC patients treated with CDKI.

As a double-edged sword, RBCs supply oxygen to tissues and organs to maintain the health and they are also associated with immuno-inflammation. Previous *in vitro* and *in vivo* studies have shown that senescent RBCs develop phosphatidylserine outgrowth on their inner cell membrane, leading to the release of reactive oxygen species. This process polarizes macrophages from the M2 to the M1 type within the inflammatory and hypoxic tumor microenvironment, while the increase of iron ions in the tumor microenvironment promotes ferroptosis.^23^ In terms of lymphocytes, RBCs not only facilitate the recruitment of circulating lymphocytes to the endothelium of the vasculature,^24^ but also enhance T cell proliferation and survival by inhibiting oxidative stress, thereby inhibiting T cell apoptosis and increasing their proliferation and survival.^45^ In addition, the profile of cytokines in erythrocyte lysates differs from that in plasma and leukocytes. For example, the level of IL-16 in RBCs is 85 times higher than that in plasma.^46^ By regulating cytokines such as IL-8 and vascular endothelial growth factor, RBCs also reduce apoptosis and stimulate the proliferation of T-lymphocytes,^47^^,^^48^ and alterations in the immuno-inflammatory microenvironment can also counteract RBCs by affecting their normal state.^49^^,^^50^ The equilibrium between immune cells and RBCs is fundamental, while disruption of this balance may give rise to changes in the immune state. It could be speculated that the index combining both immune cells and RBCs may be able to more precisely reflect the relative magnitude of immune state in the individual compared with the index from immune cells alone and act as a pragmatic biomarker in the clinical practice. Based on the above theory, our team previously first constructed RBC balanced immuno-inflammatory parameters, including LRR, NRR, and MRR, and demonstrated their effectiveness in predicting the pathologic complete response and DFS in locally advanced breast cancer patients treated with neoadjuvant chemotherapy.^25^ Following these findings, this study assessed and confirmed the prognostic value of RBC balanced immune-inflammatory parameters (LRR, NRR, MRR, and PRR) in hormone receptor-positive, HER2-negative ABC patients treated with CDKI.

Accordingly, we constructed the RBC-IMM score, and identified RBC-IMM score, rather than classical-IMM score, as an independent prognostic biomarker for both PFS and OS. In the subgroup analysis, RBC-IMM score also performed more superior than classical-IMM score in predicting PFS in both palbociclib or abemaciclib group and first line or > first line CDKI treatment for advanced disease group. Moreover, we established the clinico+RBC_index and validated its better performance over clinico_index in predicting PFS. Surprisingly, we also unveiled the association of RBC-IMM score with the CD56^+^CD16^+^ NK cells, outweighing the performance of classical-IMM score. Remarkably, the RBC-IMM score extended its predictive capability to treatment-emergent AEs, including leukopenia and possibly AST increased. Therefore, our findings provide significant implications for the value of RBC balanced features in early prediction of CDKI efficacy and toxicity, which warrants the probe into underlying mechanisms.

In order to further investigate the intrinsic pattern of RBC balanced immunophenotyping for CDKI treatment, we performed metabolomics based on RBC-IMM risk group. Through differential metabolite analysis, we discovered metabolites associated with immune system. For example, D-galactose, a metabolite inducing oxidative stress, contributes to brain inflammation and tumor progression^51^ presented high level in RBC-IMM high-risk group. Additionally, the pathway analysis showed that metabolic abnormalities existed in phosphatidylcholine synthesis, a crucial process in glycerophospholipid pathway. In this process, phosphatidylcholine, as a major component of the cell membrane and source of energy, plays a key role in the regulation of immune cells in the tumor microenvironment,^52^ which was in line with our findings that PtdCho score potentially related to CDKI efficacy and immunocyte subsets. Previous studies have found that phosphatidylcholine could be hydrolyzed into pro-inflammatory cytokines, prostaglandin E2,^53^ platelet-activating factor,^54^ and lysophosphatidic acid^55^ by phospholipase A2 and promotes the proliferation, survival and migration of tumor cells. Therefore, the metabolomic results further elucidated that RBC might impact immuno-inflammatory processes through metabolic pathways with regard to prognosis and safety for patients treated with CDKI. Since this is an exploratory research with a limited sample size, the predictive performance and intrinsic mechanisms of the metabolites need to be further validated.

In this study, we successfully addressed a crucial research gap for the prediction of CDKI efficacy by investigating the influence of various baseline RBC balanced immuno-inflammatory parameters on the prognosis and toxicity of patients treated with CDKI. The immuno-inflammatory RBC-IMM score and RBC-IMM index successfully realized early prediction, which were much more efficient than classical-IMM score and classical-IMM index. Through metabolomics analysis, the identified RBC related metabolites exhibited superior prognostic value for PFS. Our findings offer insights to the development of relevant biomarkers in large-scale clinical trials and lay foundation for potential mechanisms in future translational studies.

### Limitations of the study

The limitations of this study are as follows. First, the CDKIs in this cohort included abemaciclib and palbociclib. Notably, we conducted subgroup analysis and no significant difference in PFS was observed between the two groups. Second, this is an ambispective study with a relatively small number of patients. Further prospective studies with larger sample size are necessary to be conducted. Third, we only analyzed the baseline level of hematological parameters in this study. Nevertheless, our findings are conducive to early prediction of CDKI efficacy. As it should be, the fluctuation of parameters might occur during CDKI treatment, and therefore, further exploration is also necessary for dynamic detection.

## Resource availability

### Lead contact

Further information and requests for resources should be directed to and will be fulfilled by the lead contact, Wenjin Yin (yinwenjin@renji.com).

### Materials availability

This study did not generate new unique reagents.

### Data and code availability

• Data generated in this study have been deposited at https://www.ebi.ac.uk/metabolights/MTBLS10792.
• All original codes have been deposited in github at https://github.com/MaJiayi2024/MTBLS10792.
• Any additional information required to reanalyze the data reported in this paper is available from the lead contact upon request.

## Acknowledgments

We thank the participants of this study for their valuable contributions to the examinations and follow-up.

This study was funded by 10.13039/501100001809National Natural Science Foundation of China (No. 82173115 and 82103695), 10.13039/501100013105Shanghai Rising-Star Program (No. 22QC1400200), Shanghai Municipal Health Commission Health Industry Clinical Research Special Project (No. 202340085), Clinical Research Innovation Nurturing Fund of Renji Hospital and United Imaging (No. 2021RJLY-002), Nurturing Fund of Renji Hospital (No. PYIII20-09 and RJPY-LX-002), Shanghai Municipal Key Clinical Specialty and Beijing Foundation of Medicine Award (No. YXJL-2020-0941-0737).

## Author contributions

J.Y.M. and Y.H.W. contributed equally to this study. J.Y.M.: Conceptualization, data curation, investigation, visualization, software, writing – original draft. Y.H.W.: Methodology, writing – review, funding acquisition, and editing. Z.P.W.: Resources and funding acquisition. L.H.Z., Y.P.L., and S.G.X.: Resources. J.Z.: Data curation. J.S.L. and W.J.Y.: Conceptualization, funding acquisition, project administration, methodology, and writing – review and editing.

## Declaration of interests

The authors declare no competing interests.

## STAR★Methods

### Key resources table

**Table:** 

REAGENT or RESOURCE	SOURCE	IDENTIFIER
**Deposited data**		

Metabolomic profiling of patients	This paper	MetaboLights database: https://www.ebi.ac.uk/metabolights/MTBLS10792
Codes for screening and modeling	This paper	Github: https://github.com/MaJiayi2024/MTBLS10792

**Software and algorithms**		

R (version 4.2.2)	The R Foundation	https://www.r-project.org/
STATA Statistics SE 16	StataCorp LP, College Station	https://www.stata.com

### Experimental model and study participant details

#### Ethical statement

This study was reviewed and approved by the Independent Ethics Committee of Renji Hospital, School of Medicine, Shanghai Jiao Tong University (approval number, KY2022-097-B) and registered with ClinicalTrials.gov (NCT05795335).

#### Patients

This study ambispectively collected records of patients who received CDKI (palbociclib or abemaciclib) with endocrine therapy from April 2018 to June 2023 in Renji Hospital, School of Medicine, Shanghai Jiao Tong University. Data collected before 25 December 2022 were derived from the electronic medical records retrospectively and the consent was waived. Data after that date were collected prospectively with signed informed consent. The patients were prospectively followed up until November 2023. This study was approved by the Independent Ethics Committee of Renji Hospital, School of Medicine, Shanghai Jiao Tong University (approval number, KY2022-097-B), registered with ClinicalTrials.gov (NCT05795335) and presented according to Reporting Recommendations for Tumor Marker Prognostic Studies guidelines.^56^ Laboratory and radiological assessments were conducted regularly during the treatment of CDKI.

### Method details

#### Data collection

Baseline characteristics included age at CDKI initiation (<65 or ≥65), menstrual status (pre- or post-menopause), *de novo* stage IV (yes or no), presence of liver metastasis (yes or no), number of metastatic site (≤1 or >1), line of CDKI for advanced disease (1^st^, 2^nd^ or ≥3^rd^), previous chemotherapy for advanced disease (yes or no) and endocrine combination partner (fulvestrant or other).

The baseline complete blood count was collected within three months prior to the application of CDKI. The absolute neutrophil, lymphocyte, monocyte, platelet and RBC count were recorded. The NLR was calculated as neutrophil count/lymphocyte count. The LMR was counted as lymphocyte count/monocyte count. PLR was defined as platelet/lymphocyte count, while LRR, NRR, MRR and PRR were estimated as the count of lymphocyte, neutrophil, monocyte and platelet divided by RBC count, respectively.

#### Laboratory examination

The absolute counts of circulating immunocyte subsets were examined by the method of flow cytometry using the BD Multitest IMK Kit (a four-color direct immunofluorescence reagent kit) at the Department of Clinical Laboratory, Renji Hospital, School of Medicine, Shanghai Jiao Tong University, including CD3^+^CD4^+^ T cells, CD3^+^CD8^+^ T cells, CD56^+^CD16^+^ NK cells, and total NK cells.

#### Serum sample preperation

A total of 31 serum samples of the enrolled patients were collected before their CDKI application. Two milliliters of peripheral venous blood were drawn. After 1-2 hours standing, the samples were centrifuged at 1500 rpm, 4°C for 15 min, and then the supernatant was dispensed into a new tube and stored at -80°C.

#### Liquid chromatography-tandem mass spectrometry (LC-MS/MS) analysis

According to the procedure, 80 μL of serum sample was transferred to an eppendorf (EP) tube. After the addition of 320 μL of extract solution (methanol: acetonitrile = 1:1, containing isotopically-labelled internal standard mixture), the sample was incubated for 1 h at -40°C to precipitate proteins. Then the sample was centrifuged at 12000 rpm for 15 min at 4°C. The resulting supernatant was transferred to a fresh glass vial for analysis. LC-MS/MS analyses were performed using an UHPLC system (UltiMate 3000 UHPLC Systems, Thermo Fisher Scientific) with a UPLC BEH Amide column (2.1 mm × 100 mm, 1.7 μm) coupled to Orbitrap Exploris 120 mass spectrometer (Orbitrap MS, Thermo). The raw data were converted to the mzXML format using ProteoWizard and processed with an in-house program, which was developed using R and based on XCMS, for peak detection, extraction, alignment, and integration.

### Quantification and statistical analysis

Cutoff points for continuous variables were decided through R package ‘maxstat’. In terms of PFS, the cutoff value for NLR, LMR, and PLR was 3.16, 2.21, and 154.67 respectively and the corresponding value for LRR, NRR, MRR, and PRR was 0.24, 0.96, 0.15, and 57.82 respectively. The cutoff value for the metabolites was also obtained through the method described above. Based on the HR of PFS in the univariate analysis, patients with high risk of progression or death were assigned a score of 1 and those with low riskwere assigned a score of 0. The classical-IMM score was the sum of NLR, LMR and PLR score, while RBC-IMM score was the sum of LRR, NRR, MRR and PRR score. In metabolomics analysis, a score of 0 for RBC-IMM was categorized as the low-risk group, while a score of ≥ 1 as the high-risk group. The phosphatidylcholine (PtdCho) score was the sum of the risk score for different structures of PtdCho, including PC(22:4(7Z,10Z,13Z,16Z)/18:3(6Z,9Z,12Z)), PC(20:3(8Z,11Z,14Z)/15:0), PC(20:2(11Z,14Z)/15:0) and PC(20:1(11Z)/15:0), high level of which were assigned a score of 1 for each. PtdCho score of 0 or 1 was determined as low level and ≥2 implied high level.

Differences between the categorical variables were compared through chi-square test or Fisher's precision probability test. We examined the relationship between the continuous variables of immunocyte subpopulations and immuno-inflammatory parameters through Pearson correlation coefficient and conducted the linear fit through the R package ‘corrplot’ and ‘ggpubr’. PFS was defined as the time from the first dose of CDKI to the first occurrence of radiological or pathological disease progression per Response Evaluation Criteria In Solid Tumors (RECIST) version 1.1^57^ or death from any cause. OS was defined as the time from the first dose of CDKI to death due to any cause. The mPFS and mOS were estimated via a life table, while median follow-up time was analyzed by the reverse Kaplan-Meier method. The log-rank test and Cox proportional hazards regression analysis for univariate and multivariate analysis were performed for time-to-event variables. Time-dependent ROC were plotted through R package ‘timeROC’.

Their corresponding HR were presented with 95% CIs. AEs were assessed during study period and graded according to National Cancer Institute Common Terminology Criteria for Adverse Events (NCI CTCAE) version 5.0.^58^ Logistic regression analyses were used to derive ORs with 95% CIs to evaluate the correlations of RBC-IMM score and classical-IMM score with AEs.

To build the predictive model, the data were randomly split into a training set and a testing set at an 6:4 ratio. Based on the LASSO algorithm and 10-fold cross validation, the top 5 variables were selected. Nomograms were established to display the predicted probabilities of PFS event. The ROC analysis and DCA were conducted to assess the predictive performance of the different models by using the R package ‘survivalROC’ and ‘ggDCA’. Both AUC of ROC and C-index values of 0.6–0.7, 0.7–0.8, and 0.8–1 indicated low, medium, and high predictive accuracy, respectively.^59^^,^^60^ Calibration curve, operated by R package ‘foreign’, was applied to evaluate the difference between the observed and predicted value. The AIC was utilized to assess the performance of the model. A lower AIC value indicated a better-fitting model.

LC-MS/MS enables fingerprinting of metabolites. In order to visualize group separation and identify significantly changed metabolites, supervised OPLS-DA was applied to obtain the variable projected importance (VIP). The differential metabolites were determined by VIP>1.5 and P<0.05, together with the fold change (FC) >1.2 or <0.8. The metabolites were annotated through HMDB and illustrated with the Interactive Pathways Explorer v3 (iPath 3, https://pathways.embl.de). The pathways enrichment and pathway analysis were visualized by the KEGG (https://www.kegg.jp) database and the Small Molecule Pathway Database (SMPDB, https://smpdb.ca) to find the key pathways related to differential metabolites.

Statistical analysis was performed using STATA Statistics SE 16 (StataCorp LP, College Station, TX, USA) and R software (version 4.2.2). P value less than 0.05 was considered statistically significant. The False Discovery Rate (FDR) procedure was utilized in multivariable analysis to reduce the false positive rates.

### Additional resources

This study was registered with ClinicalTrials.gov (https://clinicaltrials.gov).
