# Supplementary material for: RBC balanced immuno-inflammatory signatures identify advanced breast cancer patients on CDK4/6 inhibitors at increased risk of progression and death
Source: iScience. 2025 May 9;28(6):112620. doi: 10.1016/j.isci.2025.112620 (PMC12152653; doi:10.1016/j.isci.2025.112620)
Supplement: Document S1. Figures S1–S7 and Tables S1–S7 [file mmc1.pdf]

iScience, Volume *28*

Supplemental information

RBC balanced immuno-inflammatory signatures identify advanced breast cancer patients on CDK4/6 inhibitors at increased risk of progression and death

Jiayi Ma, Yaohui Wang, Ziping Wu, Liheng Zhou, Yanping Lin, Shuguang Xu, Jie Zhang, Jingsong Lu, and Wenjin Yin

Supplementary materials

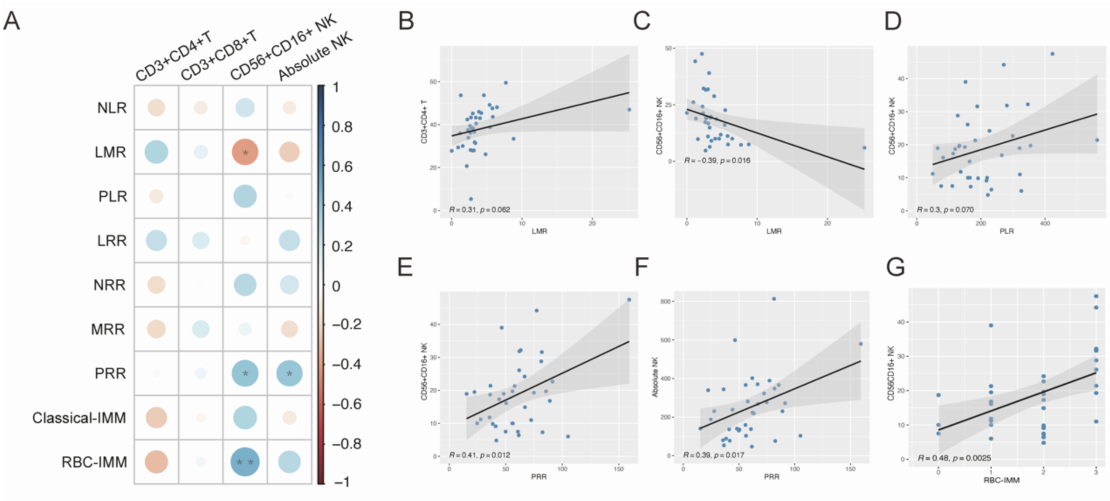

Figure S1. The correlations of immuno-inflammatory parameters and immunocytes. (A) Correlation between immuno-inflammatory parameters and immunocytes calculated by Pearson correlation analysis (*P < 0.05, **P < 0.01). Larger dots represent higher correlation coefficient. Orange dots indicate negative correlation, while blue ones represent positive correlation. (B-G) Correlations between (B) LMR and CD3+CD4+ T cells, (C) LMR and CD56+CD16+ NK cells, (D) PLR and CD56+CD16+ NK cells, (E) PRR and CD56+CD16+ NK cells, (F) PRR and absolute NK cells, (G) RBC-IMM score and CD56+CD16+ NK cells.

Abbreviations: LMR, lymphocyte to monocyte ratio; NK, natural killer; PLR, platelet to lymphocyte ratio; PRR, platelet red blood cell ratio; RBC, red blood cell; IMM, immuno-inflammatory.

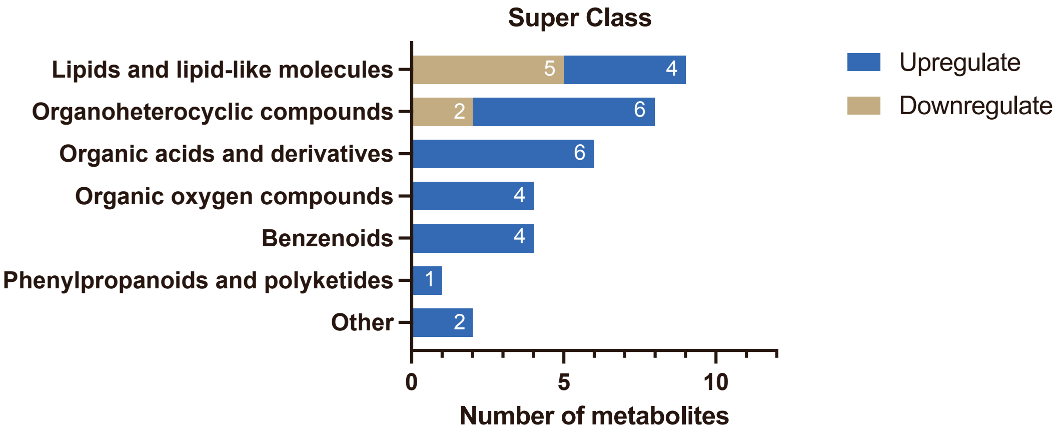

Figure S2. Metabolites significantly upregulated or downregulated in RBC-IMM high-risk group relative to low-risk group according to the HMDB.

Abbreviations: RBC, red blood cell; IMM, immuno-inflammatory; HMDB, human metabolome database.

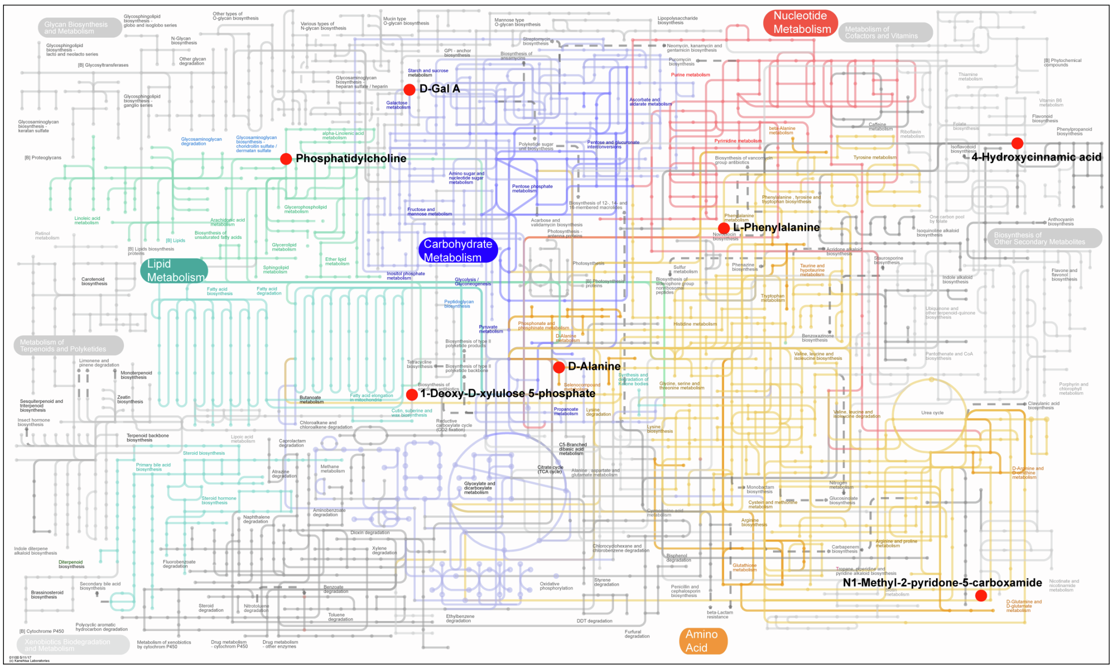

Figure S3. Differential expression metabolites (red dots) according to RBC-IMM risk group (high-risk vs. low-risk) overlaid on human metabolic reference map (KEGG: hsa01100) using Interactive Pathways Explorer v3 (iPath 3). Pathways involving amino acid metabolism (yellow lines), nucleotide metabolism (red lines), carbohydrate metabolism (blue lines) and lipid metabolism (green lines) were highlighted.

Abbreviations: RBC, red blood cell; IMM, immuno-inflammatory.

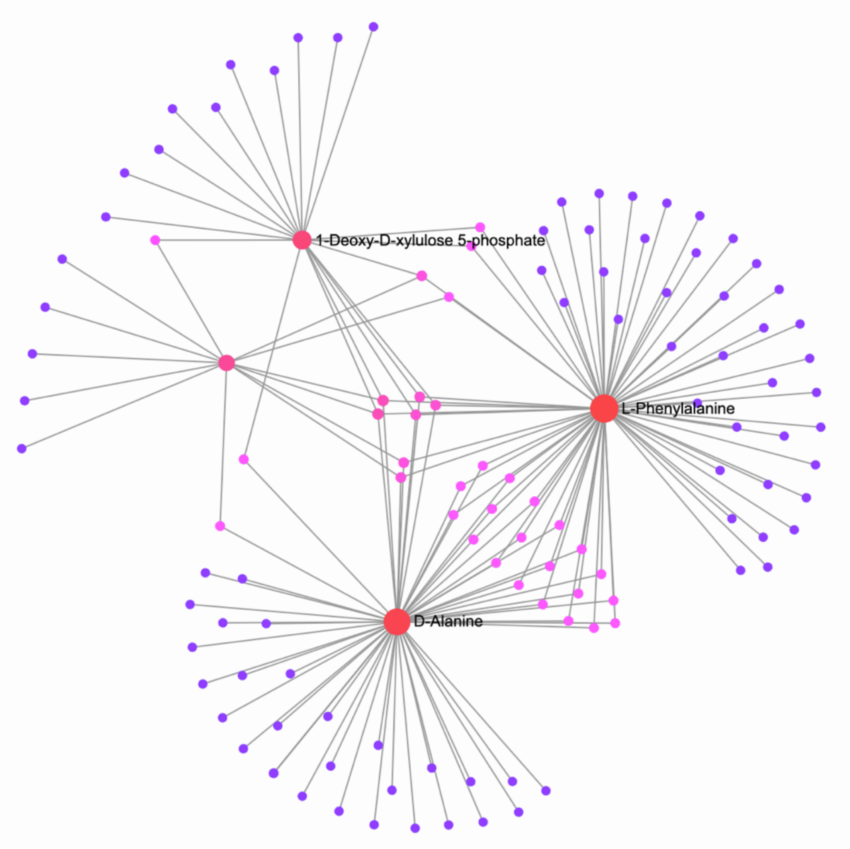

Figure S4. Network plotting based on differentially expressed metabolites according to RBC-IMM risk group (high-risk vs. low-risk), in which red circles denote strong correlations, pink circles indicate moderate correlations and purple circles represent weak correlations, with the size of the circles being proportional to the strength of the correlations.

Abbreviations: RBC, red blood cell; IMM, immuno-inflammatory.

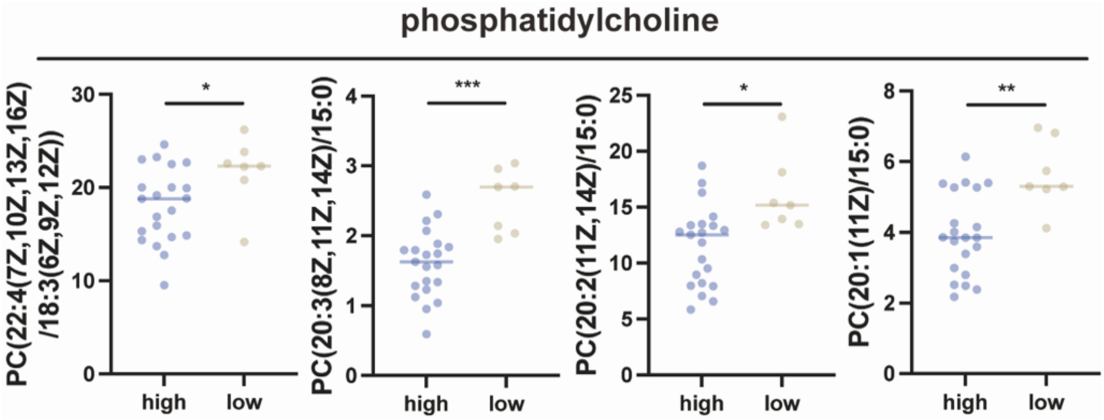

Figure S5. Phosphatidylcholines differentially expressed in different RBC-IMM risk groups (high-risk vs. low-risk). The statistical analysis was carried out by Student's t test. Error bars represent means ± SD, $^*P < 0.05$, $^{**}P < 0.01$, $^{***}P < 0.001$

Abbreviations: RBC, red blood cell; IMM, immuno-inflammatory.

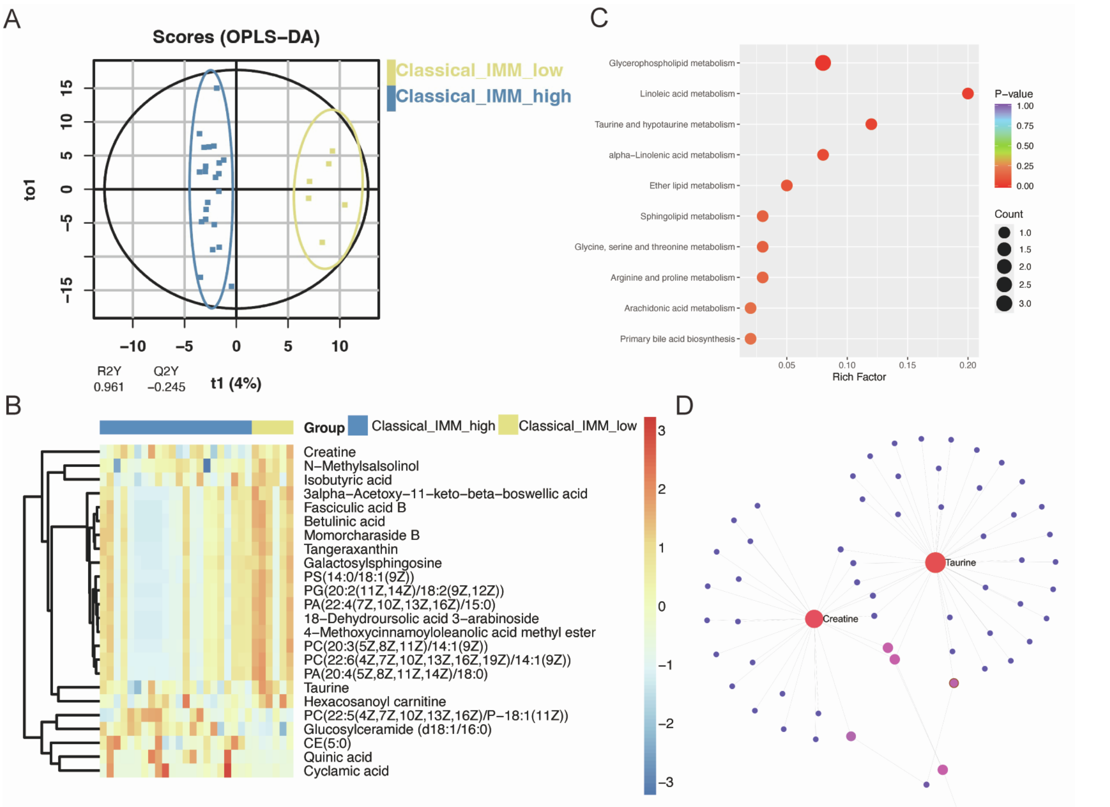

Figure S6. Metabolomics analysis according to classical-IMM score. (A) OPLS-DA score plot based on classical-IMM low-risk (yellow) and high-risk (blue) group. (B) Differentially expressed metabolites clustering heatmap according to classical-IMM risk group (high-risk vs. low-risk). (C) Metabolic pathway analysis according to KEGG based on differentially expressed metabolites. (D) Network plotting based on (B).

Abbreviations: OPLS-DA, orthogonal projection to latent structures discriminant analysis; IMM, immuno-inflammatory; cla, classical; KEGG, Kyoto Encyclopedia of Genes and Genomes.

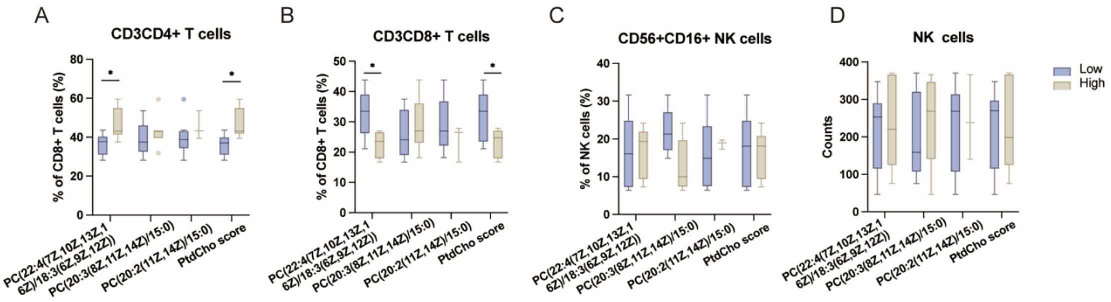

Figure S7. Boxplot of (A) CD3+CD4+ T cells, (B) CD3+CD8+ T cells, (C) CD56+CD16+ T cells and (D) NK cells in different PtdChos and PtdCho score groups. PC(20:2(11Z,14Z)/15:0) was excluded because of the small sample size in one subgroup. Blue boxes indicated low level of the metabolites and the yellow boxes represented high level of metabolites. The statistical analysis was carried out by Student's t test. Error bars represent means ± SD, *P < 0.05.

Abbreviations: PtdCho, phosphatidylcholine; NK, natural killer.

Table S1. Prognostic value of clinicopathological characteristics and immuno-inflammatory parameters in PFS for ABC patients treated with palbociclib or abemaciclib

Characteristics	Comparison	Palbociclib, N=50 HR (95% CI)	P value	Abemaciclib, N=50 HR (95% CI)	P value
Age	≥65 vs. <65	0.35 (0.16-0.74)	**0.006**	0.77 (0.36-1.65)	0.497
Menopausal status	Post- vs. pre-	0.58 (0.29-1.13)	0.107	0.52 (0.25-1.12)	0.095
Visceral involvement	Yes vs. No	1.49 (0.78-2.87)	0.228	2.17 (0.88-5.35)	0.092
Liver involvement	Yes vs. No	1.68 (0.91-3.09)	0.095	2.34 (1.12-4.90)	**0.024**
Number of metastatic sites	>1 vs. 1	1.68 (0.92-3.09)	0.093	1.56 (0.75-3.25)	0.235
Line of CDKI for advanced disease	>1st vs. 1st	1.78 (0.96-3.31)	0.068	3.70 (1.40-9.74)	**0.008**
Previous chemotherapy for advanced disease	Yes vs. No	1.40 (0.75-2.62)	0.288	2.10 (0.99-4.47)	0.053
Baseline NLR	High vs. Low	2.01 (1.05-3.86)	**0.036**	2.28 (1.09-4.76)	**0.028**
Baseline LMR	High vs. Low	0.75 (0.35-1.59)	0.456	0.27 (0.10-0.70)	**0.007**
Baseline PLR	High vs. Low	1.26 (0.68-2.35)	0.467	1.66 (0.80-3.47)	0.175
Baseline LRR	High vs. Low	0.30 (0.13-0.67)	**0.003**	0.75 (0.32-1.77)	0.517
Baseline NRR	High vs. Low	2.20 (0.96-5.03)	0.062	1.70 (0.73-4.00)	0.221
Baseline MRR	High vs. Low	2.02 (1.04-3.91)	**0.038**	3.91 (1.49-10.24)	**0.006**
Baseline PRR	High vs. Low	1.29 (0.71-2.33)	0.409	2.21 (1.06-4.63)	**0.035**
Baseline classical-IMM score	3 vs. 2 vs. 1 vs. 0	1.24 (0.93-1.65)	0.136	1.49 (1.07-2.07)	**0.019**
Baseline RBC-IMM score	3 vs. 2 vs. 1 vs. 0	1.65 (1.19-2.28)	**0.003**	2.74 (1.60-4.67)	**0.003**

Abbreviations: PFS, progression-free survival; CDKI, cyclin-dependent kinase 4/6 inhibitor; ABC, advanced breast cancer; NLR, neutrophil to lymphocyte ratio; LMR, lymphocyte to monocyte ratio; PLR, platelet to lymphocyte ratio; LRR, lymphocyte to red blood cell ratio; NRR, neutrophil to red blood cell ratio; MRR, monocyte to red blood cell ratio; PRR, platelet red blood cell ratio; RBC, red blood cell; IMM, immuno-inflammatory; HR, hazard ratio; CI, confidence interval.

Table S2. Predictive value of different immuno-inflammatory scores in PFS for patients receiving 1st and >1st line CDKI therapy for advanced disease

Characteristics	Comparison	1st line, N=42 HR (95% CI)	P value	>1st line, N=48 HR (95% CI)	P value
Baseline classical-IMM score	3 vs. 2 vs. 1 vs. 0	1.82 (1.21-2.72)	**0.004**	1.18 (0.91-1.53)	0.202
Baseline RBC-IMM score	3 vs. 2 vs. 1 vs. 0	2.17 (1.27-3.73)	**0.005**	1.52 (1.15-2.01)	**0.004**

Abbreviations: PFS, progression-free survival; CDKI, cyclin-dependent kinase 4/6 inhibitor; IMM, immuno-inflammatory; RBC, red blood cell; HR, hazard ratio; CI, confidence interval.

Table S3. Predictive value of clinicopathological characteristics and immuno-inflammatory parameters in OS for all patients

Characteristics	Comparison	HR (95% CI)	P value (FDR)
Age	≥65 vs. <65	0.57 (0.21-1.55)	0.272 (0.314)
Menopausal status	Post- vs. pre-	0.90 (0.35-2.32)	0.833 (0.833)
Visceral involvement	Yes vs. No	3.18 (0.93-10.84)	0.065 (0.098)
Liver involvement	Yes vs. No	2.71 (1.15-6.40)	**0.023 (0.043)**
Number of metastatic sites	>1 vs. 1	3.33 (1.28-8.67)	**0.014 (0.032)**
Line of CDKI for advanced disease	>1st vs. 1st	1.22 (0.51-2.91)	0.655 (0.702)
Previous chemotherapy for advanced disease	Yes vs. No	1.78 (0.77-4.14)	0.181 (0.226)
Baseline NLR	High vs. Low	4.58 (1.84-11.40)	**0.001 (0.005)**
Baseline LMR	High vs. Low	0.42 (0.16-1.10)	0.077 (0.105)
Baseline PLR	High vs. Low	3.99 (1.34-11.87)	**0.013 (0.032)**
Baseline LRR	High vs. Low	0.33 (0.12-0.90)	**0.030 (0.050)**
Baseline NRR	High vs. Low	-	**-**
Baseline MRR	High vs. Low	2.98 (1.24-7.17)	**0.015 (0.032)**
Baseline PRR	High vs. Low	4.60 (1.78-11.86)	**0.002 (0.008)**
Baseline classical-IMM score	3 vs. 2 vs. 1 vs. 0	1.90 (1.29-2.81)	**0.001 (0.005)**
Baseline RBC-IMM score	3 vs. 2 vs. 1 vs. 0	4.00 (2.20-7.30)	**<0.001 (<0.001)**

Abbreviations: OS, overall survival; CDKI, CDK4/6 inhibitor; NLR, neutrophil to lymphocyte ratio; LMR, lymphocyte to monocyte ratio; PLR, platelet to lymphocyte ratio; LRR, lymphocyte to red blood cell ratio; NRR, neutrophil to red blood cell ratio; MRR, monocyte to red blood cell ratio; PRR, platelet red blood cell ratio; RBC, red blood cell; IMM, immuno-inflammatory; HR, hazard ratio; CI, confidence interval, FDR, false discovery rate.

Table S4. Multivariate analysis of OS in all patients

Characteristics	HR (95% CI)	P value (FDR)
Age		
⩾65 vs. <65	0.45 (0.15-1.37)	0.159 (0.398)
Liver involvement		
Yes vs. No	1.10 (0.43-2.82)	0.848 (0.848)
Line of CDKI treatment		
>1st vs. 1st	1.20 (0.47-3.06)	0.708 (0.848)
Baseline classical-IMM score		
3 vs. 2 vs. 1 vs. 0	1.23 (0.79-1.91)	0.362 (0.636)
Baseline RBC-IMM score		
3 vs. 2 vs. 1 vs. 0	3.67 (1.74-7.74)	**0.001 (0.005)**

Abbreviations: OS, overall survival; CDKI, CDK4/6 inhibitor; RBC, red blood cell; IMM, immuno-inflammatory; FDR, false discovery rate; FDR, false discovery rate.

Table S5. Adverse events of hematology in 69 patients and biochemistry in 68 patients

Adverse Event	All grades	Grade 3	Grade 4
Leukopenia	52 (75.36)	16 (23.19)	1 (1.45)
Neutropenia	56 (81.16)	17 (24.64)	1 (1.45)
Thrombocytopenia	22 (31.88)	2 (2.90)	1 (1.45)
Anemia	15 (21.74)	3 (4.35)	-
AST increased	17 (25.00)	-	-
ALT increased	10 (14.71)	-	-
Blood creatinine level increased	17 (25.00)	-	-

Abbreviations: AST, aspartate aminotransferase; ALT, alanine aminotransferase.

Table S6. Differential metabolites of RBC-IMM groups (high vs. low)

Index	Fold_Change	P-value	FDR	VIP	Type
Threonic acid	1.62268814	0.0177	0.55835769	1.91991702	up
Indoxyl sulfate	2.0120724	0.037	0.62251875	1.9304205	up
s2-Hydroxy-3-methylbutyric acid	1.6152152	0.00573	0.425475	1.7626753	up
N-a-Acetyl-L-arginine	1.66394875	0.0436	0.62251875	1.52010145	up
1,3-Dihydro-(2H)-indol-2-one	2.08242383	0.0375	0.62251875	1.73838791	up
7-Methylguanine	1.32899962	0.0335	0.62251875	1.59894589	up
L-Phenylalanine	1.28758652	0.0259	0.58814483	1.88472711	up
DL-Tyrosine	1.91346599	0.00909	0.425475	2.13922477	up
3-Methoxy-4-hydroxyphenylethyleneglycol sulfate	1.99004846	0.0221	0.55835769	1.89316335	up
1-(beta-D-Ribofuranosyl)-1	1.67483771	0.0077	0.425475	1.7859222	up
4-Hydroxy-3-methylbenzoic acid	2.82130297	0.00863	0.425475	3.28995496	up
SM(d18:1/14:0)	0.71480408	0.0289	0.62251875	1.9312637	down
PC(22:4(7Z,10Z,13Z,16Z)/18:3(6Z,9Z,12Z))	0.8294898	0.0473	0.62251875	1.72062488	down
Galactinol	4.35412381	0.0222	0.55835769	2.07757225	up
PC(20:3(8Z,11Z,14Z)/15:0)	0.640127	9.08E-04	0.425475	2.88656928	down
2-Methyl-5-(1-propenyl)pyrazine	1.34612976	0.0311	0.62251875	2.07067242	up
PC(20:2(11Z	0.71618463	0.013	0.48463333	2.18209309	down
1,2,3-Trihydroxybenzene	1.58997577	0.0214	0.55835769	1.59809739	up
2-Phenylacetamide	2.00093564	0.00857	0.425475	2.25381123	up
D-GalA	1.6380399	0.0132	0.48463333	1.83549698	up
Demethylated antipyrine	0.71094259	0.00207	0.425475	2.16645126	down
4-Hydroxycinnamic acid	1.95026298	0.0233	0.56178889	1.70261452	up
PC(20:1(11Z)/15:0)	0.70091992	0.0028	0.425475	2.43713416	down
Parabanic Acid	1.39188964	0.0134	0.48463333	1.58657147	up
6-Hydroxy-1H-indole-3-acetamide	0.73441773	0.00915	0.425475	1.8136026	down
D-Alanine	1.29203295	0.00779	0.425475	2.55923357	up
N1-Methyl-2-pyridone-5-carboxamide	2.02084916	0.0405	0.62251875	1.60864856	up
1-Deoxy-D-xylulose 5-phosphate	2.36204084	0.0122	0.48463333	2.2912007	up
Linoleamide	1.52735713	0.0346	0.62251875	1.61796764	up
Benzaldehyde	1.37359754	0.0214	0.55835769	2.24843182	up
ar-Artemisene	5.00055801	0.0368	0.62251875	2.02317016	up
Ethylmalonic acid	1.38647977	0.00389	0.425475	1.82151425	up
Halosulfuron-methyl	2.58237113	0.00186	0.425475	2.04630282	up
5-Acetamidovalerate	2.2713947	0.00576	0.425475	1.70528434	up

Table S7. Differential metabolites of classical-IMM groups (high vs. low)

Index	Fold_Change	P-value	FDR	VIP	Type
Taurine	0.73422405	0.0227	0.64599231	2.07778255	down
Quinic acid	3.71065557	0.00332	0.64599231	1.90943606	up
N-Methylsalsolinol	0.78678415	0.0029	0.64599231	1.7650215	down
Hexacosanoyl carnitine	0.66672436	0.0426	0.8946	2.44520187	down
Creatine	0.68131031	0.0226	0.64599231	1.73176493	down
PC(22:5(4Z,7Z,10Z,13Z,16Z)/P-18:1(11Z))	1.63731324	0.00981	0.64599231	1.82749861	up
Isobutyric acid	0.68917587	0.0443	0.90122812	2.03483754	down
3alpha-Acetoxy-11-keto-beta-boswellic acid	0.43339173	0.0233	0.64599231	1.90190147	down
CE(5:0)	1.39130571	0.00981	0.64599231	1.52456208	up
Cyclamic acid	9.11066112	0.0142	0.64599231	1.9832006	up
PC(20:3(5Z,8Z,11Z)/14:1(9Z))	0.42452609	0.0279	0.6727	1.67392013	down
PC(22:6(4Z,7Z,10Z,13Z,16Z,19Z)/14:1(9Z))	0.40420987	0.0249	0.64599231	1.86411664	down
Momorcharaside B	0.45725488	0.0147	0.64599231	1.51905842	down
Fasciculic acid B	0.45569696	0.0216	0.64599231	1.52733826	down
Glucosylceramide (d18:1/16:0)	1.84872908	0.00691	0.64599231	2.71190078	up
18-Dehydroursolic acid 3-arabinoside	0.4450158	0.0228	0.64599231	1.52052881	down
Tangeraxanthin	0.45017171	0.0195	0.64599231	1.65296186	down
PG(20:2(11Z,14Z)/18:2(9Z,12Z))	0.43742046	0.0183	0.64599231	1.66408484	down
Galactosylsphingosine	0.49133472	0.0366	0.8216069	1.80963639	down
Betulinic acid	0.47117929	0.0235	0.64599231	1.61150353	down
PA(20:4(5Z,8Z,11Z,14Z)/18:0)	0.42516613	0.0305	0.709125	2.28364654	down
PS(14:0/18:1(9Z))	0.42202439	0.0182	0.64599231	1.72238448	down
PA(22:4(7Z,10Z,13Z,16Z)/15:0)	0.43281464	0.0232	0.64599231	1.59414696	down
4-Methoxycinnamoyloleanolic acid methylester	0.441109	0.0217	0.64599231	1.55721543	down
